# Elevated expression of Nodal and YAP1 is associated with poor prognosis of gastric adenocarcinoma

**DOI:** 10.1007/s00432-016-2188-2

**Published:** 2016-06-20

**Authors:** Ping Li, Dan Sun, Xiaoting Li, Yingjian He, Wenhui Li, Jing Zhao, Ying Wang, Huan Wang, Yan Xin

**Affiliations:** 1 Laboratory of Gastrointestinal Onco-Pathology, Cancer Institute and General Surgery Institute, The First Affiliated Hospital of China Medical University, 155 Nanjing North Street, Heping District, Shenyang, 110001 Liaoning Province China; 2 Key Laboratory of Carcinogenesis and Translational Research (Ministry of Education), Peking University Cancer Hospital & Institute, No. 52 Fucheng Road, Haidian District, Beijing, 100142 China

**Keywords:** Nodal, YAP1, Gastric adenocarcinoma, Prognosis

## Abstract

**Purpose:**

To investigate the correlation between Nodal and YAP1 expression and the clinicopathological characteristics of gastric adenocarcinoma (GAC).

**Methods:**

Quantitative real-time RT-PCR, Western blot, and immunohistochemistry were performed to measure Nodal and YAP1 mRNA and protein in 20 fresh frozen samples and 220 paraffin-embedded GAC tissues with their paired non-tumor mucosa (PNTM). The prognostic values of Nodal and YAP1 were evaluated in 161 GAC patients using univariate and multivariate analyses.

**Results:**

Both mRNA and protein expression of Nodal and YAP1 were significantly increased in GAC compared to PNTM (*P* < 0.05). Immunohistochemistry showed that Nodal was more highly expressed in 56.4 % GAC samples compared to PNTM; additionally, Nodal expression correlated with depth of tumor invasion, lymph node metastasis, and distant metastasis (all *P* < 0.05). There was no association between Nodal and YAP1 in GAC (*P* = 0.171). Kaplan–Meier analysis showed that the outcome of Nodal-high patients was significantly worse than those with low Nodal expression (*χ* ^2^ = 30.452, *P* < 0.001). Cox multivariate regression showed that high Nodal expression was an independent risk factor affecting the prognosis of GAC patients (*P* = 0.000, RR = 2.976). Furthermore, patients with tumors in which both Nodal and YAP1 were expressed at high levels had the worst prognosis.

**Conclusions:**

Elevated Nodal expression is a marker of poor prognosis in gastric cancer. Patient outcome is further worsened if Nodal and YAP1 are both expressed in the same tumor. The datas we present here suggest that the inhibition of Nodal signaling may represent a new therapeutic strategy for the treatment of gastric adenocarcinoma.

## Introduction

Nodal, a member of the transforming growth factor-β (TGF-β) superfamily, regulates many diverse cellular processes, including the growth and differentiation of embryonic stem cells and the plasticity and invasiveness of malignant tumor cells. As such, Nodal helps regulate the pluripotency of embryonic stem cells and cancer cells (James et al. 2005). Nodal signaling also plays a critical role during the development of vertebrates, echinoderms, and protostomes. During early vertebrate development, Nodal is necessary and sufficient for endomesoderm induction and patterning of anteroposterior (AP), dorsoventral (DV), and left–right (LR) axes. During echinoderm development, Nodal is important for determining DV and LR asymmetry. In contrast, in protostomes, Nodal signaling is only involved in establishing LR asymmetry (Röttinger et al. 2015). While Nodal is an essential molecule early in development, it is thought to be largely absent from adult tissues; the few exceptions include some adult stem cell populations and some highly dynamic reproductive tissues (Aykul et al. 2015). Nodal signaling is regulated, at least in part, by secreted proteins Lefty and Cerberus. During development, Nodal exerts its functions both by regulating its own expression and via unilateral activation of downstream genes, such as Lefty and Pitx. In turn, Lefty and Pitx regulate expression of several additional genes, many of which have roles in proliferation and differentiation (Soukup et al. 2015). For example, one study showed that exposure of tumor cells to an hESC microenvironment containing Lefty resulted in decreased Nodal expression, colony forming ability, and tumorigenicity (Postovit et al. 2008). Additionally, Cerberus, another Nodal inhibitor that functions by preventing the interaction between Nodal and its receptor, profoundly suppresses migration, invasion, and colony formation ability of breast cancer cells (Aykul et al. 2015). Studies such as these suggest that approaches to decrease Nodal expression may have some therapeutic value. This is particularly relevant since several earlier studies have established that Nodal is up-regulated in various cancers, including breast cancer (Kirsammer et al. 2004), melanoma (Strizzi et al. 2015), endometrial cancer (Cruz et al. 2015), and pancreatic cancer (Kong et al. 2015). Nodal appears to play many roles during oncogenesis as well; indeed, Nodal has been associated with increased tumor angiogenesis, invasion, and metastasis.

YAP1 is a transcriptional coactivator of the Hippo pathway with roles in tumorigenesis. Since it regulates tumor cell proliferation and apoptosis, YAP1 has been described as a candidate oncogene (Sudol 1994). Several studies have reported increased expression of Nodal and YAP1 in many tumor types, including melanoma, liver cancer, breast cancer, ovarian cancer, and glioma. This increased expression in cancer suggests that Nodal and YAP1 might play important roles in tumor proliferation, angiogenesis, invasion, and metastasis.

Importantly, previous studies have reported cross talk between components of the Hippo and TGF-β pathways, both at the level of intracellular signaling and transcriptional regulation (Grannas et al. 2015). As one example in endothelial cells, TGF-β induces nuclear localization of a Smad2/3/4 complex, which activates expression of Snail, Twist1, and Slug, key transcription factors required for epithelial–mesenchymal transition (EMT). YAP1 interacts with this complex, and loss of YAP1 disrupts the TGF-β-mediated up-regulation of Snail, Twist1, and Slug (Zhang et al. 2014). These findings highlight the potential importance of cross talk between these two pathways for cellular processes. Despite this, to date, potential roles for Nodal and YAP1 have not been reported in gastric adenocarcinoma (GAC). In this study, we measured mRNA and protein expression of both Nodal and YAP1 in GAC and paired non-tumor mucosa (PNTM). We examined possible correlation between expression of these factors and several clinicopathological variables. The data we present here suggest that inhibition of Nodal signaling may represent a new therapeutic strategy for the treatment of gastric adenocarcinoma.

## Materials and methods

### Samples and clinicopathological data

Two hundred and twenty surgically resected gastric adenocarcinoma (GAC) specimens and paired non-tumor mucosa (PNTM) (obtained at a location greater than 5 cm away from the edge of the primary tumor) were collected at the First Affiliated Hospital of China Medical University. Specimens were fixed in 10 % neutral formalin. Conventional wax baits were cut consecutively into 4 μm sections and then subject to conventional H&E staining to determine pathological diagnosis. Twenty additional fresh frozen specimens from postoperative GAC and corresponding PNTM were collected and preserved at −80 °C for qRT-PCR and Western blot. None of the patients had received radiotherapy or chemotherapy prior to surgery. None of the patients received adjuvant or palliative therapy either. Clinical and pathological data for all patient samples used in the study are listed in Table 2. Informed consent was obtained from the Ethics Committee in the First Affiliated Hospital of China Medical University.

### RNA extraction and quantitative real-time reverse transcriptase PCR (qRT-PCR)

Total RNA was isolated from 20 tumor/non-tumor paired tissue samples using the EASYspin Plus kit (Aidlab Biotechnologies, Beijing) according to the manufacturer’s instructions. RNA quantity and quality were analyzed with Nanodrop 1000 (Thermo Fisher Scientific). Isolated RNA was reverse-transcribed to cDNA using the PrimeScript^®^ RT reagent kit (TaKaRa, Dalian, China) according to the manufacturer’s protocol. Primers to measure the expression of genes of interest were designed using Prime5 software. Primer sequences are as follows: Nodal—Sense 5′-ACATCATCCGCAGCCTACA-3′, anti-sense 5′-AGCCCATGCCAGATCCTC-3′, YAP1—Sense 5′-TACGATACAAGGCTGTTAGAGAG-3′, anti-sense 5′-TTGAGATGCATGCTTTGCATAC-3′; GAPDH—sense 5′-GAAGGTGAAGGTCGGAGTC-3′, and anti-sense 5′-GAAGATGGTGATGGGATTTC-3′.

Each PCR was prepared in a total volume of 10 µl, which included each primer, diluted cDNA templates, and SYBR^®^ Premix TaqTM II (TaKaRa, Dalian, China). Cycling conditions for qRT-PCR were as follows: 95 **°**C for 30 s followed by 40 cycles of 95 **°**C for 5 s and annealing at 58.5 **°**C for 30 s. Melting curves were generated by 95 **°**C for 15 s, 60 **°**C for 30 s, and 95 **°**C for 15 s. Data were calculated using the 2-ΔΔCt method. The mRNA level of each sample was normalized to that of GAPDH mRNA, and each cDNA sample was run in triplicate.

### Protein extraction and Western blot

Protein was harvested from samples using lysis buffer containing phenylmethylsulfonylfluoride (PMSF) at 4 **°**C. Total extracted proteins were quantified using a BCA protein assay kit (ComWin Biotech, Beijing, China). Samples were boiled at 95 °C for 5 min, and then, 50 μg of protein was electrophoretically separated using 10 % SDS-PAGE gels (Invitrogen). Following electrophoresis, samples were transferred to a polyvinylidene difluoride (PVDF) membrane via wet transfer. PVDF membranes were blocked with 5 % nonfat dry milk in Tris–phosphate buffer containing 0.05 % Tween 20 (TBS-T) for 1 h at room temperature, and then incubated with primary antibodies at room temperature overnight. The following primary antibodies were used in this study: rabbit anti-Nodal polyclonal antibody (working dilution 1:1000, MILLIPORE), rabbit anti-YAP1 monoclonal antibody (1:1000, Abcam, Britain), and β-actin (1:1000, ZhongShan-Golden Bridge, Beijing), which was used as an internal loading control. Membranes were then incubated with horseradish peroxidase-conjugated anti-mouse or anti-rabbit secondary antibodies (1:1000, ZhongShan-Golden Bridge, Beijing) and visualized using an enhanced chemiluminescence (ECL) detection system. Blots were visualized using an ECL Kit (ComWin Biotech, Beijing, China) and quantified using ImageJ software. Results are expressed as fold change compared to the control values. All experiments were performed at least three times.

### Tissue microarray and immunohistochemistry

Two hundred and twenty GAC and paired normal mucosa samples were fixed in 10 % formalin, embedded in paraffin, and cut into 4 μm-thick sections. All samples were evaluated by two experienced pathologists, who confirmed the diagnoses and marked various target lesions. Six blocks of tissue microarray containing 220 gastric adenocarcinomas and their corresponding normal gastric mucosa were constructed using microarrayer (USA). The six blocks of tissue microarray were then cut to yield 4-μm serial sections and placed on glass slides using an adhesive-tape transfer system for immunohistochemistry.

Nodal and YAP1 protein expression were measured using two-step immunohistochemistry. Rabbit anti-Nodal polyclonal antibody (working dilution 1:50) and rabbit anti-YAP1 monoclonal antibody (working dilution 1:50) were purchased from MILLIPORE and Abcam, respectively. The DAB kit was purchased from Fuzhou Maixin Company (Fuzhou, China). The PV-9000 kit was obtained from Beijing Zhongshan Goldenbridge Biotechnology Company (Beijing, China). Tissue microarray slides were deparaffinized in xylene and hydrated with alcohol before being placed in 3 % H_2_O_2_ methanol blocking solution. This was then followed by heat-induced antigen retrieval. The slides were incubated with primary antibodies overnight at 4 °C, stained with the PV-9000 detection system, and counterstained with hematoxylin. All procedures were carried out according to the manufacturer’s instructions. For negative controls, sections were treated with 0.01 M phosphate-buffered saline instead of primary antibodies.

### Assessment of immunohistochemistry staining

Nodal or YAP1 positivity was defined as samples displaying clear brown granules either in the cytoplasm or nucleus. The expression of Nodal and YAP1 was determined by assigning proportion and intensity scores to both tumor cells and adjacent gastric mucosa epithelial cells. This type of staining analysis has been previously described (Vandeputte et al. 2002). The proportion score was assigned based on the proportion of positive cells (0, none; 1, ≤10 %; 2, 11–25 %; 3, 26–50 %; 4, >50 %). The intensity score was assigned based on comparison to YAP1-positive or Nodal-positive internal controls (0, none; 1, weak; 2, intermediate; 3, strong). The total score for Nodal or YAP1 expression, ranging from 0 to 12, was the product of the proportion and intensity scores. The expression was categorized as negative (0), (−); low (score 1–3), (1+); intermediate (score 4–6), (2+); or high (score 7–12), (3+). According to the above-mentioned criteria, samples scoring 0–1+ were considered the low-expression group, and samples scoring 2+ to 3+ were considered the high-expression group for subsequent statistical analysis.

### Statistical analysis

Categorical data have been described using frequencies and percentages. Continuous data have been presented as means with standard deviations for normally distributed data. Statistical analysis was performed using SPSS 17.0 software. Either *Chi-square* test or Fisher’s exact test was used to differentiate the rates of different groups. Time-to-event data were estimated by the Kaplan–Meier method and analyzed with the log-rank test. The cumulative overall survival rates were calculated using life table techniques, illustrated by Kaplan–Meier plots. The multivariable analysis model was fit using a Cox proportional hazards regression model using SPSS 17.0. The enter method was used to determine a final Cox model. All statistical analyses were two-sided, and significance was assigned at *α* = 0.05.

## Results

### Nodal and YAP1 mRNA expression are significantly up-regulated in GAC

**Fig. 1:**
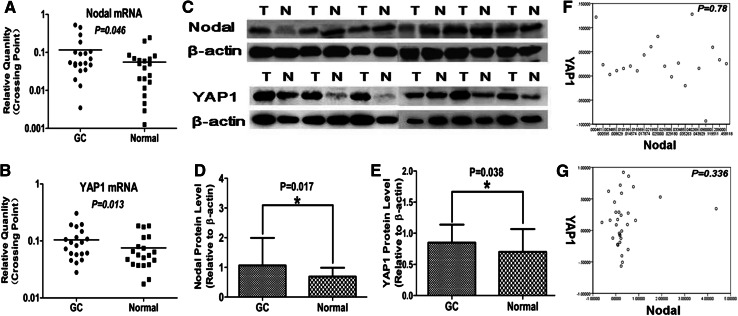
Expression of Nodal and YAP1 mRNA and protein in GAC. Nodal mRNA (**a**) and YAP1 mRNA (**b**) were significantly up-regulated in GAC compared with PNTM (*P* < 0.05); similar results were found when examining protein expression (**c**–**e**). However, there was no correlation between Nodal and YAP1 expression at either the mRNA (**f**) or protein (**g**) level

To determine expression levels of Nodal and YAP1, we performed qRT-PCR using RNA isolated from GAC samples and from adjacent normal gastric mucosa. Nodal mRNA was significantly higher in GAC tissue compared to paired normal mucosa (*P* = 0.046). Similarly, YAP1 mRNA expression was also elevated in GAC specimens compared to paired normal mucosa (*P* = 0.013) (Fig. 1; Table 1).

**Table 1:** Nodal and YAP1 mRNA and protein expression in GC and paired normal mucosa

Groups	*n*	Nodal expression	*P*	YAP1 expression	*P*
mRNA			**0.046**		**0.013**
GC	20	0.126 ± 0.177		0.105 ± 0.069	
Normal	20	0.056 ± 0.063		0.075 ± 0.052	
Protein			**0.017**		**0.038**
GC	32	1.0699 ± 0.9241		0.8494 ± 0.2893	
Normal	32	0.6929 ± 0.2965		0.7012 ± 0.3667	

Bold values indicates that statistical significance of *P* value is less than 0.05

### Nodal and YAP1 protein expression are significantly up-regulated in GAC

We next performed Western blot from GAC samples and their matched normal tissue to determine protein expression of Nodal and YAP1. Consistent with our mRNA analysis, we found significantly increased protein expression of both Nodal (*P* = 0.017) and YAP1 (*P* = 0.038) in GAC tissues compared to matched normal mucosa (Fig. 1; Table 1).

### Expression of Nodal and YAP1 are not correlated in GAC

Since both Nodal and YAP1 are elevated in GAC samples compared to normal mucosa, we next determined if there was any correlation between expressions of these two factors. We found no correlation between Nodal and YAP1 at either the mRNA (*P* = 0.78) or protein (*P* = 0.336) level in either GAC or paired normal tissue expression (Fig. 1).

### High Nodal protein expression is associated with poor clinicopathological variables in GAC

**Fig. 2:**
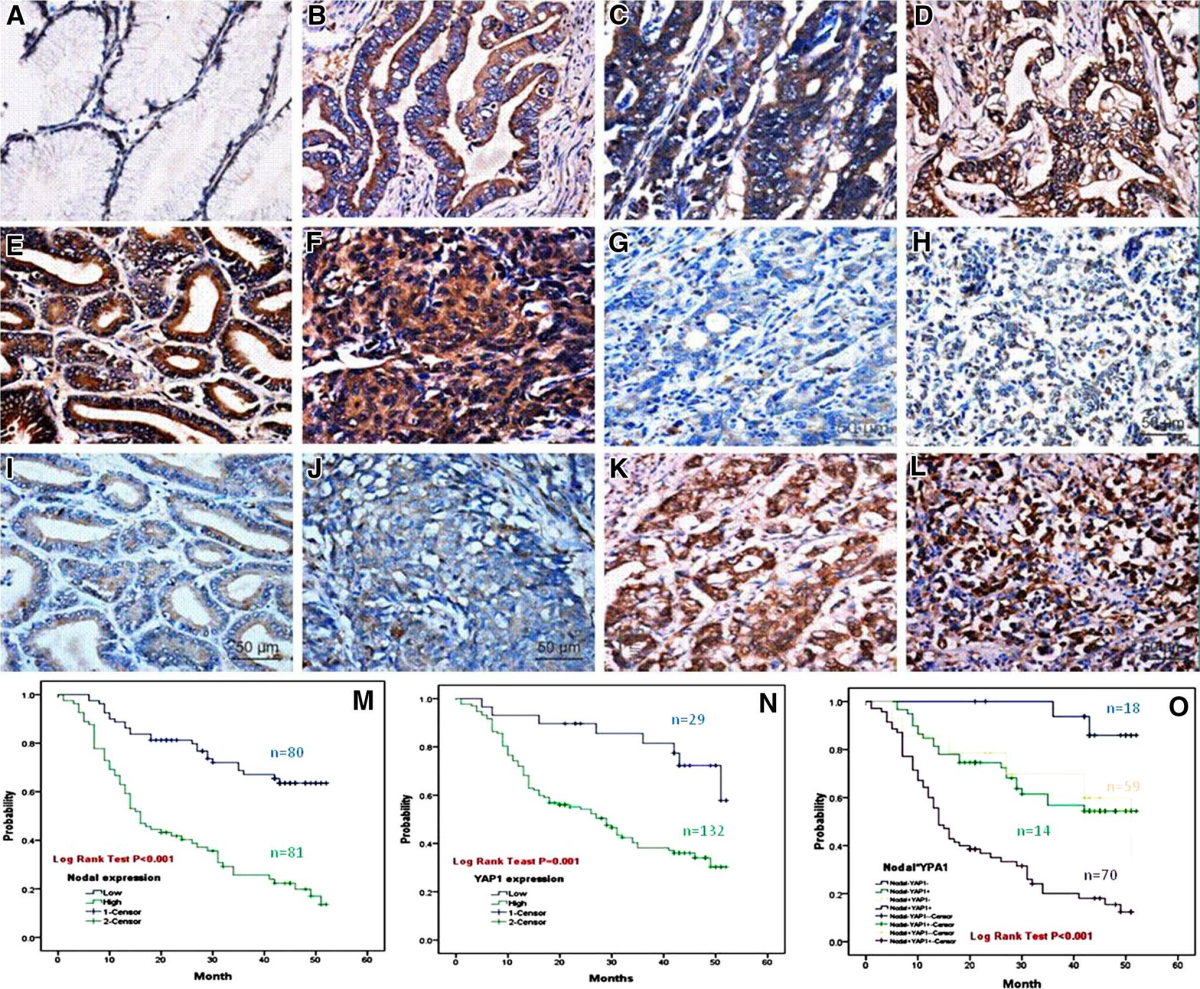
Correlation between expression of Nodal and YAP1 protein and prognosis of patients with GAC. Compared with the PNTM (**a**), Nodal expression was significantly up-regulated in GAC (**b**–**f**). Nodal expression (**e**–**h**) was not correlated with YAP1 expression (**i**–**l**) in the same case of GAC (**e** vs. **i**, **f** vs. **j**, **g** vs. **k**, **h** vs. **l**). Two-step IHC ×400. Kaplan–Meier analyses of the effects of Nodal and YAP1 expressions on patient survival in GAC showed that overall survival for patients with high Nodal expression was significantly worse than that with low expression (*P* = 0.000, **m**). Overall survival for patients with YAP1 high-expression was significantly worse than that in the YAP1 low-expression group (*P* = 0.001, **n**). Furthermore, there was an interaction between Nodal and YAP1 expression on GAC outcome, as patients with tumors with high expression of both Nodal and YAP1 had the worst overall prognosis (**o**)

We next wanted to determine if there was any correlation between Nodal expression and clinicopathological variables in GAC. Immunohistochemistry showed that Nodal protein was expressed both in the cytoplasm and nucleus of GAC samples. A total of 56.4 % (124/220) of GAC samples stained positive for Nodal, in contrast to the only 4.3 % (8/186) of paired normal tissue showing a positive stain (*P* < 0.01). Furthermore, Nodal expression in GAC correlated with depth of tumor invasion, lymph node metastasis, distant metastasis, and TNM staging (all *P* < 0.005) (Fig. 2; Table 2).

**Table 2:** Correlation between Nodal and YAP1 expression and clinicopathological features in GC

Variable	*n*	Nodal expression		HPR (%)	*χ* ^2^	*P* value
		Low	High			
Age (year)					3.696	0.055
≤55	82	43	39	47.6		
>55	138	54	84	60.9		
Gender					0.018	0.892
Female	67	30	37	55.2		
Male	153	67	85	56.2		
Borrmann’s types					0.26	0.61
I + II	30	12	18	60.0		
III + IV	189	85	104	55.0		
WHO’s histology types					8.930	0.159
Papillary. Ade.	8	3	5	62.5		
Tubular Ade.						
Well Diff.	14	9	5	35.7		
Moderately Diff.	74	36	38	51.4		
Poorly Diff.	99	35	64	64.6		
Un-differentiated car.	3	1	2	66.7		
Signet ring cell Car.	7	5	2	28.6		
Mucinous Ade.	15	8	7	46.7		
Lauren’s types					0.735	0.692
Intestinal	80	38	42	53.5		
Diffuse	102	42	60	58.8		
Mixed	38	17	21	55.3		
Depth of invasion					8.899	**0.003**
T1 + T2	36	24	12	33.3		
T3 + T4	184	73	111	60.3		
Ln metastasis					19.669	**<0.001**
N0	60	41	19	31.7		
N1–3	160	56	104	65.0		
Distant metastasis					23.815	**<0.001**
M0	167	89	78	46.7		
M1	53	8	45	84.9		
TNM staging					26.416	**<0.001**
I + II	71	49	22	31		
III + IV	149	48	101	67.8		
YAP1 expression					1.8712	0.171
Low	41	22	19	46.3		
High	179	75	104	58.1		

*HPR* high positive rate, *Histol.* histological, *Papi.* papillary, *ade.* adenocarcinoma, *diff.* differentiation, *Undiff.* undifferentiated, *Car* carcinoma, *SRC* signet ring cell cancer, *Ln.* lymph node

Bold values indicates that statistical significance of *P* value is less than 0.05

### High Nodal expression is associated with poor prognosis in GAC

In this study, a total of 161 cases were followed up for 52 months after surgery. The median survival time was 34 ± 4.79 months. A total of 88 deaths occurred during follow-up, and all causes of death were cancer-related. Kaplan–Meier survival curves were plotted, and a log-rank test was used to evaluate the effect of high Nodal expression on patient outcome. The mean survival time of patients in the Nodal-high group was 23.58 ± 1.98 months, significantly shorter than in the Nodal-low group (40.90 ± 1.88 months) (*P* < 0.001). These results indicate that high Nodal expression is associated with poor prognosis in gastric adenocarcinoma (Fig. 2m; Table 3).

**Table 3:** Univariate and multivariate analyses of different prognostic factors in 161 patients with GC

Variable	Univariate analysis^a^			Multivariate analysis^b^	
	*n*	Mean survival (months)	*P* value	HR (95 % CI)	*P* value
Borrmann’s types			0.289	1.113 (0.646–1.985)	0.663
I + II	22	29.55			
III + IV	139	32.62			
WHO’s histology types			0.638	1.096 (0.913–1.317)	0.324
Papillary. Ade.	6	32.50			
Tubular Ade.					
Well Diff.	11	33.80			
Moderately Diff.	56	35.29			
Poorly Diff.	70	29.71			
Un-differentiated car.	3	35.67			
Signet ring cell car.	5	16.60			
Mucinous Ade.	10	26.07			
Lauren’s types			0.354	1.055 (0.747–1.492)	0.760
Intestinal	56	35.11			
Diffuse	77	31.41			
Mixed	28	28.13			
Depth of invasion			0.001	1.886 (0.895–3.975)	0.095
T1 + T2	28	43.96			
T3 + T4	133	29.54			
Ln metastasis			0.001	0.450 (0.208–0.974)	0.043
N0	43	41.06			
N1–3	118	28.64			
TNM staging			<0.001	6.999 (2.754–17.786)	<0.001
I + II	52	46.15			
III + IV	109	25.23			
Nodal protein expression			<0.001	2.487 (1.546–4.001)	<0.001
Low	80	40.90			
High	81	23.58			
YAP1 protein expression			0.001	2.506 (1.195–5.257)	0.015
Low	29	44.90			
High	132	29.22			
Nodal and YAP1 expression			<0.001		
Nodal^−^YAP1^−^	18	50.30			
Nodal^−^YAP1^+^	59	37.35			
Nodal^+^YAP1^−^	14	39.38			
Nodal^+^YAP1^+^	70	21.51			

*Ade* Adenocarcinoma, *Diff*. Differentiated, *Car*. Carcinoma, *Ln.* lymph node

^a^Log-rank test; ^b^ Cox regression model

### Patients with GAC tumors expressing both Nodal and YAP1 exhibit the worst overall prognosis

To evaluate potential interaction between Nodal and YAP1 on GAC prognosis, patients were divided into four groups according to their Nodal and YAP1 expression status. The groups consisted of patients with tumors that were Nodal^−^YAP1^−^, Nodal^−^YAP1^+^, Nodal^+^YAP1^−^, and Nodal^+^YAP1^+^. Kaplan–Meier analysis showed that overall survival for patients with YAP1 high-expression was significantly worse than in patients with low YAP1 expression (*P* = 0.001, Fig. 2n). Patients with low expression of both Nodal and YAP1 (Nodal^−^YAP1^−^) demonstrated the best outcome. In contrast, patients with high expression of both proteins (Nodal^+^YAP1^+^) demonstrated the worst outcome (Fig. 2o; Table 3.)

## Discussion

Nodal is a member of the TGF-β superfamily that regulates many important aspects of early development, including germ layer specification and axis patterning. While its expression in many adult tissues is down-regulated, it has been shown to be re-expressed in cancer cells, where it likely promotes tumorigenesis. This claim is supported by data showing that down-regulation of Nodal significantly reduces tumorigenicity of several cancer cell line models (Lee et al. 2010; Quail et al. 2012; Topczewska et al. 2006). YAP1 is a member of the Hippo signaling pathway, which regulates organ size by coordinating cellular proliferation and apoptosis. Overexpression of YAP1 has been shown to correlate with disease progression and poor prognosis of gastric cancer. Previous studies have reported cross talk between components of the Hippo and TGF-β pathways, both at the level of intracellular signaling and transcriptional regulation (Grannas et al. 2015). Despite this, interplay between these two pathways in gastric adenocarcinoma has not been thoroughly examined. To gain insight into the role of Nodal and YAP1 in gastric cancer, we examined their expression in a panel of patient tumor samples and matched normal tissue. We found that elevated expression of either Nodal or YAP1 is associated with several clinicopathological variables. Additionally, patients with tumors expressing high levels of both Nodal and YAP1 displayed the worse prognosis. The data we present here suggest that inhibition of the Hippo and TGF-β pathways in gastric adenocarcinoma may have some therapeutic benefit.

We first examined mRNA and protein expression of Nodal and YAP1 in a panel of gastric adenocarcinoma patient samples and matched normal tissue. We measured significant overexpression of Nodal and YAP1 at both the mRNA and protein levels in tumors compared to normal tissue. These findings suggest that these factors and their respective signaling pathways might be drivers of disease progression. This is supported by earlier work showing that YAP1 expression correlates with progression, metastasis, and poor prognosis in patients with gastric carcinoma (Hu et al. 2014). Moreover, Nodal has been found to be up-regulated in various cancers, including breast cancer (Kirsammer et al. 2004), endometrial cancer (Cruz et al. 2015), pancreatic cancer (Kong et al. 2015), and melanoma (Strizzi et al. 2015). The melanoma study is particularly interesting since the authors showed no expression of Nodal in normal skin but increased nodal expression in approximately 60 % of invasive melanoma cases. These data correlate with the findings we present here and support the fact that Nodal expression is associated with advanced disease state and overall prognosis.

We expanded on these initial observations by examining the potential correlation between Nodal and YAP1 expression and several clinicopathological variables. Nodal expression correlated with depth of tumor invasion, lymph node metastasis, and distant metastasis. Furthermore, we found that either high Nodal or YAP1 expression served as an independent risk factor for GAC prognosis. Previous work from our group has already established that YAP1 also correlates with these same clinicopathological variables (Hu et al. 2014). Importantly, we discovered that patients with tumors in which both Nodal and YAP1 were expressed at high levels had the worse overall prognosis. These data suggest that these two factors or their respective signaling pathways may cooperate to promote disease progression. Since we did not detect significant correlation between expression of Nodal and YAP1 themselves, it is possible that the Hippo and TGF-β pathways interact downstream at the signal transduction level to enhance disease pathogenesis. Additional work is required to better understand the nature of the cross talk between these two pathways.

The work we present here is not without its limitations. Most notably, the data we present here do not address the reason why gastric adenocarcinomas expressing both Nodal and YAP1 at high levels have the worse overall prognosis. Second, sample size is limited, which prevents us from drawing vast conclusions; additional studies with more samples would enhance the work we present here. Third, follow-up information for all patients after surgery was not available; this may have introduced bias into our analysis. Finally, the data we present are largely correlative. Follow-up studies in either cell lines or animal models in which levels of Nodal or YAP1 could be manipulated would help establish a possible causal role for either of these factors in disease progression.

In summary, we show that Nodal and YAP1 are increased in gastric adenocarcinomas compared to normal tissue. Elevated Nodal expression is a marker of poor prognosis in gastric cancer. Patient outcome is further worsened if Nodal and YAP1 are both expressed in the same tumor. The data we present here suggest that inhibition of Nodal signaling may represent a new therapeutic strategy for the treatment of gastric adenocarcinoma.
